# A 30-year bibliometric analysis of the literature in the disciplines of orthopedics, surgery, or oncology on chondrosarcoma from 1993 to 2023

**DOI:** 10.1097/MD.0000000000037182

**Published:** 2024-02-09

**Authors:** Selçuk Yilmaz, Mehmet Kurt

**Affiliations:** a Kutahya Health Sciences University, Faculty of Medicine, Department of Orthopedics and Traumatology, Kütahya, Turkey.

**Keywords:** bibliometric analysis, chondrosarcoma, Web of Science

## Abstract

**Background:**

A thorough bibliometric analysis of publications published in the field of chondrosarcoma research has not yet been performed using the Web of Science database, especially for publications published between 1993 and 2023. This study, with a focus on the fields of orthopedics, surgery, and oncology, aims to fill this knowledge gap by providing a thorough analysis of current knowledge in the field of chondrosarcoma.

**Methods:**

In this bibliometric study, a literature search was performed using the Web of Science database to find all publications on chondrosarcoma. A bibliometric software program was used for data visualization and analysis (opensource visualization application, Vosviewer). The Web of Science Core Collection data used for this retrospective bibliometric study, which covers the period from January 1993 to September 2023, revealed interesting trends in chondrosarcoma research.

**Results:**

As the most popular fields of study, orthopedics, surgery, and oncology account for a sizable portion of publications. A noteworthy increase in research output from 2014 to 2023, accounting for 41.74% of the papers, reflects the thriving research environment. The leading countries for publication were China, Japan, and the United States, demonstrating cross-border cooperation in chondrosarcoma research. Their contributions were highlighted by their important affiliations with institutions such as Harvard University, Leiden University, and China Medical University Taiwan. A thorough keyword mapping analysis also highlighted research priorities and encouraged interdisciplinary cooperation. The field’s scholarly importance and ongoing relevance are highlighted by the study’s high citation count (30,076) and highly cited articles.

**Conclusion:**

Overall, this study offers crucial insights into the development and collaborative nature of the chondrosarcoma research landscape and its long-lasting influence on academic research and clinical practice.

## 1. Introduction

Chondrosarcoma is a slow-growing tumor that refers to a diverse group of primary bone tumors that are distinguished by the presence of hyaline cartilaginous neoplastic tissue. Chondrosarcoma is the second most common type of primary bone cancer.^[1,2]^ Most cases of chondrosarcoma occur in adults.^[1]^ Approximately 3 new cases of chondrosarcoma have been reported annually per 10^6^ people.^[3]^ They are categorized as primary and secondary based on their developmental characteristics; central, peripheral, and juxtacortical (periosteal) based on where they were found in the bone; conventional based on histological subgroups (grade 1–3), and dedifferentiated based on the cell types that make up the periosteum, mesenchyme, and transparent tissue.^[4]^ The predominant conventional subtype of chondrosarcoma is observed in >90% of the cases. Conventional chondrosarcomas can develop de novo or as a result of a benign underlying lesion, such as an osteochondroma or enchondroma.^[5]^ Most chondrosarcomas are conventional chondrosarcomas, which are typically low- to intermediate-grade tumors (grade 1 or 2) with passive clinical behavior and limited metastatic potential.^[2]^ Grade 3 conventional chondrosarcomas comprise only 5% to 10% of cases and have a high risk of metastasizing.^[6]^ The most common symptoms observed in patients are pain, dullness, and soreness localized to the tumor site.^[4]^ A favorable prognosis can be achieved with effective surgery because metastases are uncommon in most chondrosarcoma cases. However, because high-grade chondrosarcomas are resistant to radiation and chemotherapy, thorough surgical excision is required.^[7]^ Novel therapeutic options to improve prognosis are still being determined, and the etiology of chondrosarcoma involves complex systems that are not fully known. Therefore, identifying the current developmental status and hotspots of chondrosarcoma in both orthopedic and oncological disciplines is necessary to aid researchers in gaining insight into this topic.^[1,8]^

Although a quantitative overview can be performed using a variety of techniques, including traditional reviews, systematic reviews, main path analyses, and evidence maps, only bibliometrics allow us to simultaneously assess the knowledge base and popular research topics while qualitatively and quantitatively analyzing the contributions and collaboration of authors, institutions, countries, and journals.^[9–15]^

Numerous research papers on this subject have been published owing to the recent rapid advancements in medical science and technology on chondrosarcoma in the last 30 years. As a result, it is crucial to comprehend and obtain knowledge from fresh developments and trends in chondrosarcoma development. Finding timely information on the status of a particular area of medical research remains difficult. The development of bibliometric analysis has made it possible to statistically and quantitatively visualize evidence in a particular field in accordance with data from different sources (books, journals, databases, etc). By objectively presenting the research contributions of many different countries, institutions, and authors from a large number of published studies and forecasting research hotspots and trends, bibliometric analysis has emerged as the most useful tool for describing the situation and exploring research directions. However, there has not been any bibliometric analysis of chondrosarcoma from the Web of Science (WOS) database analyzing publications between 1993 to 2023, and not much attention has been paid to estimating research hotspots. This study seeks to present a thorough analysis of the current state of knowledge and to forecast future developments in the field of chondrosarcoma since 1993 in the disciplines of orthopedics, surgery, or oncology.

## 2. Material and methods

### 2.1. Data collection

This was a retrospective bibliometric study that used data from an open single electronic database. To eliminate bias caused by database daily changes, we comprehensively downloaded all original papers from the Web of Science Core Collection (WOSCC) with a publication date constraint from January 1, 1993, to September 1, 2023, for this study. The following retrieval approach was utilized for the search: (TS = Chondrosarcoma*) (the sign “*” is a retrieval symbol for the WOS advanced retrieval and indicates that you want to look for words that come before the word “*”). Medical Subject Headings (MeSH) terms are a type of standard vocabulary that may be used to carry out ongoing analyses of frequent word sets and reflect the major subjects of literary works. The search and data analysis were performed using Clarivate Analytics’ Web of Science’s “All Databases.”

We conducted a search using the National Center for Biotechnology Information’s (NCBI) National Library of Medicine’s (NLM) search criteria (“Chondrosarcoma” [MeSH]), and since no further synonyms were found, the keywords for the WOSCC search were chosen as previously described. In the present study, only articles available in English were included in the final analysis after language constraints were applied to the literature searches and downloads.

### 2.2. Search strategy inclusion and exclusion criteria

The inclusion criteria were published records with a predominance of chondrosarcoma-related articles published between January 1, 1993, and September 1, 2023. Articles unrelated to chondrosarcoma, duplicate information, conference abstracts, letters, news articles, editorial materials, and retracted or withdrawn publications were excluded to obtain consistent and accurate data from the outcomes of various analyses based on published studies. After receiving uniform training, the reviewers independently gathered all the data by selecting articles based on their titles and abstracts and, when necessary, extracted the complete text from those publications. The arguments between the 2 researchers were discussed and settled.

There were 3860 publications published between January 1, 1993, and September 1, 2023, of which 2571 were articles. A total of 2474 of these publications were published in English. Only articles in the disciplines of orthopedics, surgery, and oncology were included in this analysis. As 1310 articles met the study’s main goals, a thorough examination was performed.

### 2.3. Graphical mappings and descriptive statistics

The following details were noted for each selected article: subject area, publication name and year, author, journal, institution, country of origin, top-cited articles, top-publishing journals, cooperating institutions, and number of citations. Graphical mapping and descriptive statistics were used to analyze the data.

The Visualization of Similarities Viewer program (VOSviewer v.1.6.19; Centre for Science and Technology Studies, Leiden University, Netherlands; accessible at https://www.vosviewer.com)^[16]^ was used to create graphical mappings of the bibliometric data.

The construction of the bibliographic networks was based on the co-authorship network, co-authorship network of countries, and co-occurrence of keywords. The minimum number of published papers by authors and the minimum number of published articles by countries have also been increased to 5 to improve the clarity of co-authorship network maps. The network maps node sizes, with larger nodes denoting higher frequency, indicated the investigated item’s frequency (keyword, author, and country). While their colors indicate the cluster to which the item belongs, the thickness of the edges is proportional to how closely the 2 nodes interact with one another.

## 3. Results

### 3.1. General information

This study included 1310 articles. The results showed that a large percentage of these were specifically related to orthopedics (321 articles, 24,504%), surgery (478 articles, 36,489%), and oncology (690 articles, 52,672%).

An examination of 1310 articles in the domains of orthopedics, surgery, and oncology from 1993 to 2023 over 3 ten-year periods uncovered interesting patterns. A large number of articles in the first 10 years (1993 to 2003) were published in 2000. The most articles were published in 2010, and the second decade (2004 to2013) made up 33.82% of the total. The most recent ten years (2014 to 2023) saw the greatest increase, accounting for 41.74% of the articles; the most productive year was 2020. This pattern reflects a vibrant and expanding research landscape in these specialist domains, with a considerable rise in scholarly contributions over the last ten years, indicating greater study interest and activity.

Additionally, the h-index, a reliable measure of academic productivity and impact, reached a significant value of 81. This indicated that 2316 work in the dataset had at least 17.37 citations each, demonstrating the breadth and impact of the body of literature that had been compiled over the time period under consideration.

A thorough analysis of the open-access status of the 1310 papers under analysis produced intriguing results. A total of 563 papers (42.977%) were categorized as “All Open Access,” demonstrating the openness of this research. Of these, 60 papers (4.580%) were labeled as “Gold-Hybrid,” while 272 articles (20.763%) were classified as “Gold” Open Access. Additionally, 146 articles (11.145%) had the designation “Free to Read,” which enhanced accessibility. 375 (28.626%) of the papers were “Green Published” indicating some kind of open access. Articles that were “Green Accepted” (2.214%) and “Green Submitted” (8.015%) constituted a smaller percentage. Notably, 57.023% of the records lacked information on whether they were Open Access, suggesting that this dataset might be more transparent and adhere to Open Access standards.

The distribution of the WOS Index was as follows in the analysis of 1310 articles: the majority (88.779%) were included in the prestigious “Science Citation Index Expanded (SCI-EXPANDED),” indicating their significant presence there. Only 8.779% were included in the “Emerging Sources Citation Index (ESCI),” indicating that they have gained some notoriety among more recent research sources. Additionally, 2.443% and 2.366% of the publications were listed in the “Book Citation Index—Science (BKCI-S)” and “Conference Proceedings Citation Index—Science (CPCI-S),” respectively, indicating that they were included in important reference categories. Finally, considering the dataset’s predominately scientific focus, only 0.076% of the publications were listed in the “Social Sciences Citation Index (SSCI),” indicating insufficient representation in the social sciences.

### 3.2. Most profilic countries, continents, affiliations, authors, and founders

Authors from 63 countries and 1466 affiliations have contributed to the literature. Examining the top publishing continents and areas in this dataset revealed some interesting patterns. With 31.603% of all publications, the United States of America took the lead and exhibited a strong presence in the chosen fields. China (People’s Republic of China) and Japan are the next largest contributors, each with approximately 12% of the articles, showing their robust research output in these fields. Europe is highly represented, with top donors, including Italy, the Netherlands, England, Germany, France, and Spain. The South Asian nation, India, also makes a significant contribution.

Asia, dominated by China, Japan, and Taiwan, is the second-largest publishing region in the world after North America, and is primarily made up of the United States of America and Canada. Europe is a large additional contributor, with various nations playing an active role. These findings highlight the international nature of research in these domains, which is dominated by North America, Asia, and Europe.

Research on chondrosarcoma has been greatly aided by the specialized departments of a wide variety of academic and medical institutions. Notably, the Department of Pathology at Leiden University Medical Center took the lead in 3.511% of all papers on the subject. Other noteworthy departments that each contributed more than 1% of the papers were those at the University of Miami School of Medicine, Department of Orthopaedic Surgery at Massachusetts General Hospital, and University College London. Additionally, the presence of the University College London Medical School, Harvard Medical School Department of Orthopaedic Surgery, emphasizes the international nature of chondrosarcoma research.

The National Natural Science Foundation of China, with 60 records and 4.580% of total funding, was the most significant factor among the 399 funders identified. Along with the United States Department of Health and Human Services, the United States National Institutes of Health also reported 58 instances, totaling 58 instances and 4.427% of the funds. The National Cancer Institute of the National Institutes of Health mentioned 27 records, constituting 2.061% of the total. The Japan Society for the Promotion of Science and the Ministry of Education, Culture, Sports, Science, and Technology had 23 records (1.756% of the total). A total of 20 projects, accounting for 1.527% of the funding, were Grants-in-Aid for Scientific Research (Kakenhi) and Taiwan’s Ministry of Science and Technology.

### 3.3. Citing analysis

A total of 30,076 times were cited in favor of chondrosarcoma research. However, after excluding self-citations, there were 23,048 citations in total. Each article from this research had an average of 22.96 citations.

Most publications (n = 88) were published in 2018, according to WOS data. Notably, 2296 citations were made for these 2018 publications. Surprisingly, 2021 had the highest number of citations, with 2265 citations indicating publications from that year.

**Figure 1.**
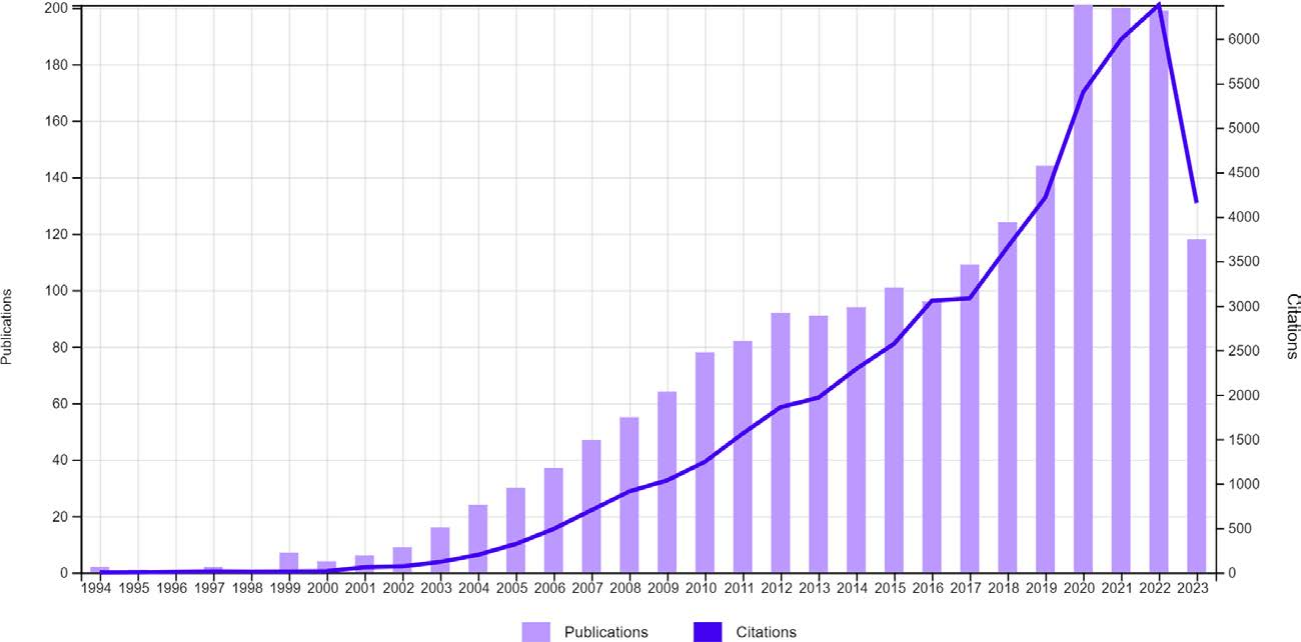
Times cited and publications over time.

The field of chondrosarcoma research has greatly benefited from 3 highly cited articles. The study by Amary et al in the Journal of Pathology (2011) that states “IDH1 (isocitrate dehydrogenase 1) and IDH2 (isocitrate dehydrigenase 2) mutations are frequent events in central chondrosarcoma and central and periosteal chondromas but not in other mesenchymal tumors” stands out with 676 citations. Another noteworthy piece is “The clinical approach towards chondrosarcoma” by Gelderblom et al, which was published by an oncologist in 2008 and received 440 citations with an impressive annual citation rate of 27.5. Finally, Damron et al‘s article “Osteosarcoma, Chondrosarcoma, and Ewing’s Sarcoma” from Clinical Orthopaedics and Related Research (2007) received 354 citations, averaging about 20.82 citations per year. These works, with a high number of citations, demonstrate their significance and enduring impact on the field of chondrosarcoma research (Table 1 and Fig. 1).

**Table 1:** Highly cited articles chondrosarcoma.

Title	Authors	Source title	Publication year	Total citations	Average per year	DOI
IDH1 and IDH2 mutations are frequent events in central chondrosarcoma and central and periosteal chondromas but not in other mesenchymal tumors	Amary et al	Journal of Pathology	2011	676	52	10.1002/path.2913
The clinical approach towards chondrosarcoma	Gelderblom et al	Oncologist	2008	440	27.5	10.1634/theoncologist.2007-0237
Osteosarcoma, chondrosarcoma, and Ewing’s sarcoma	Damron et al	Clinical Orthopaedics and Related Research	2007	354	20.82	10.1097/BLO.0b013e318059b8c9
Proton radiation therapy for chordomas and chondrosarcomas of the skull base	Hug et al	Journal of Neurosurgery	1999	344	13.76	10.3171/jns.1999.91.3.0432
Chordomas and chondrosarcomas of the cranial base – Results and follow-up of 60 patients	Gay et al	Neurosurgery	1995	311	10.72	10.1227/00006123-199505000-00001
Chondrosarcoma of bone: An assessment of outcome	Lee et al	Journal of Bone and Joint Surgery-American Volume	1999	303	12.12	10.2106/00004623-199903000-00004
Risk factors for survival and local control in chondrosarcoma of bone	Fiorenza et al	Journal of Bone and Joint Surgery-British Volume	2002	238	10.82	10.1302/0301-620X.84B1.11942
Chondrosarcoma in the United States (1973–2003): An analysis of 2890 cases from the SEER database	Giuffrida et al	Journal of Bone and Joint Surgery-American Volume	2009	227	15.13	10.2106/JBJS.H.00416
Chondrosarcoma of the base of the skull – A clinicopathologic study of 200 cases with emphasis on its distinction from chordoma	Rosenberg et al	American Journal of Surgical Pathology	1999	214	8.56	10.1097/00000478-199911000-00007
Primary chondrosarcoma of long bones and limb girdles	Bjornsson et al	Cancer	1998	208	8	

### 3.4. Co-authorship analysis between countries and organizations

**Figure 2.**
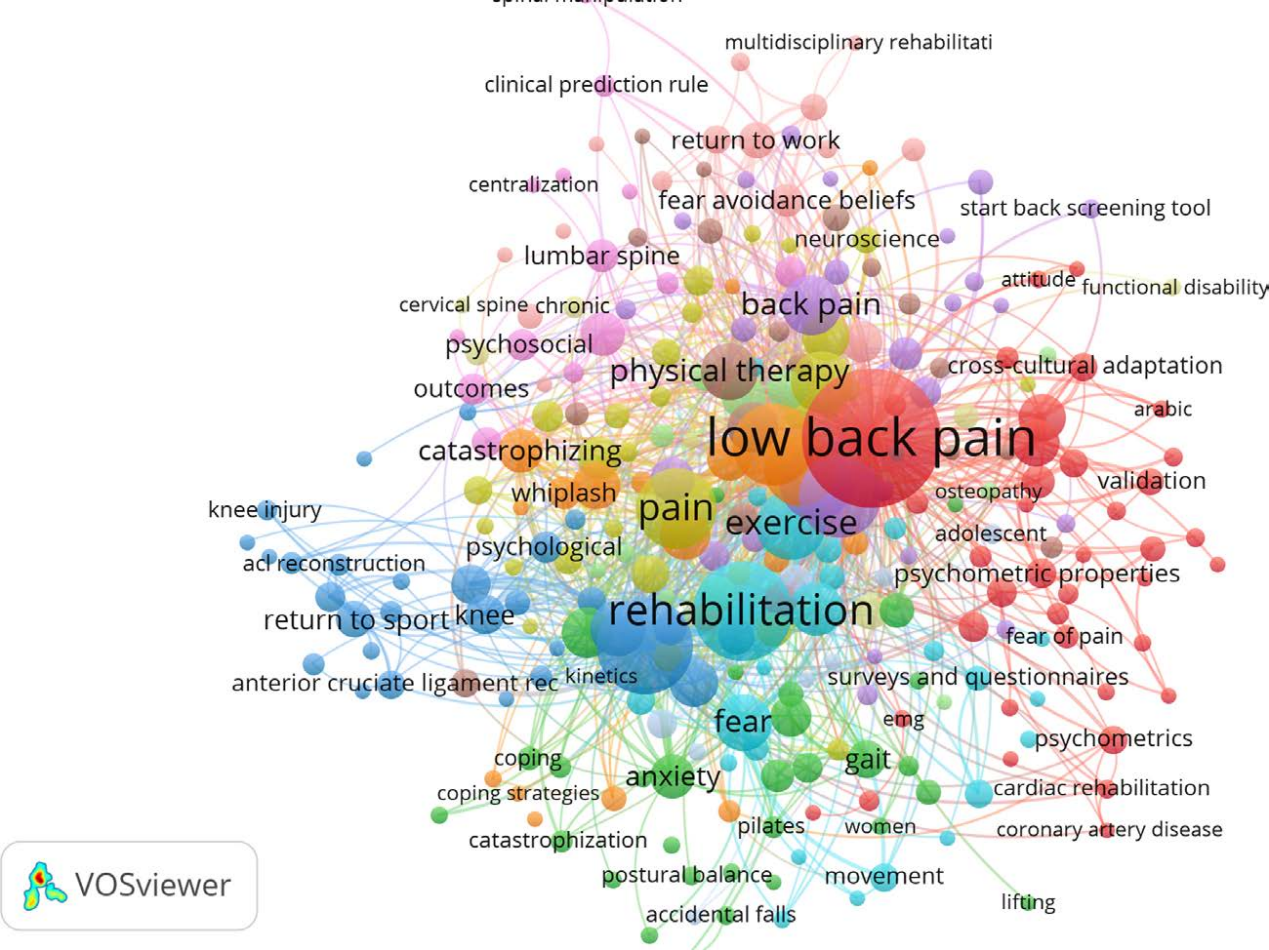
The co authorship between countries.

With a minimum document criterion of 5, a co-authorship mapping study using Vosviewer revealed that 30 out of 71 countries met the requirements. According to the number of papers, citations, and total link strength contributed by these nations, the top contributors are as follows: 410 documents, 12,318 citations, and 141 links overall, with the United States in first place. The People’s Republic of China has 169 documents, 1905 citations, and link strength of 26. Japan has made 158 documents, 2475 citations, and 33 links in total. 91 documents, 2502 citations, and a link strength of 77 were used to represent Italy. Eighty documents, 4088 citations, and a link strength of 70 were all related to the Netherlands. There were 72 documents from England with 2764 citations and a link strength of 64. The following country is Germany has 68 documents, 2414 citations, and a link strength of 54. 1 827 citations, 55 documents, and a link strength of 56. India has 51 documents, 176 citations, and a link strength of 7. Turkey has 39 documents, with 465 citations and a link strength of 16. According to their co-authorship network link strength, the number of documents, and the number of citations, these nations have contributed significantly to joint research projects (Fig. 2).

**Figure 3.**
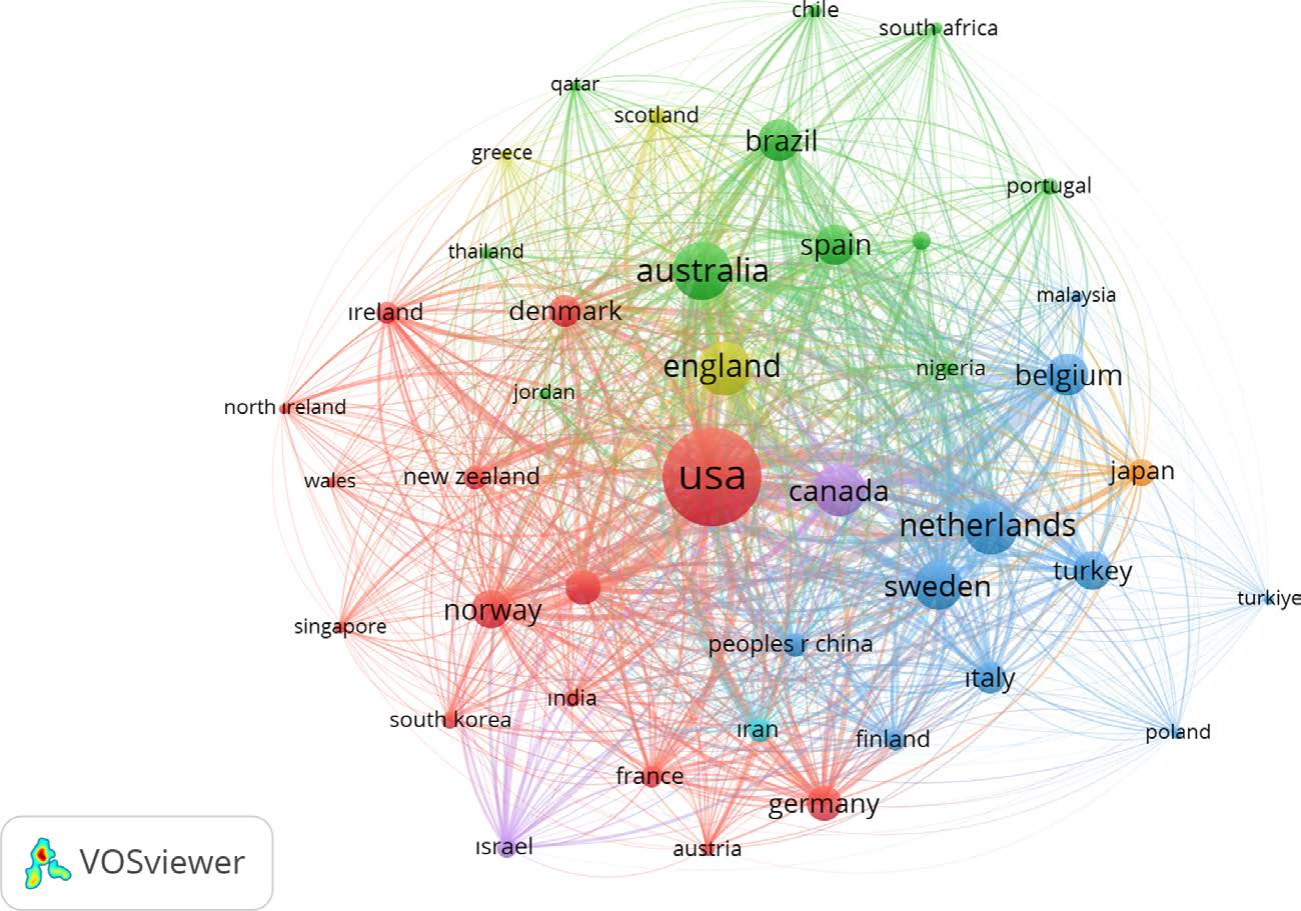
The co authorship analysis between organizations.

A minimum document threshold of 5 was used in a co-authorship mapping analysis using Vosviewer, and 101 out of 1572 organizations met the requirements. In terms of research output, citations, and overall link strength, a few of these institutions stood out as significant contributors representing various parts of the world. The top organization was Leiden University, which is based in the Netherlands and produced 58 documents, racked 3537 citations, and displayed a strong total link strength of 25. China Medical University, a Chinese institution, came in second place, with 26 documents, 838 references, and a strong link strength of 50. In the US, 26 documents were produced by the Mayo Clinic, the University of Miami, and the University of Texas MD Anderson Cancer Center. The Mayo Clinic received 740 citations with a link strength of 15, the University of Miami received 933 citations, and the MD Anderson Cancer Center received 599 citations with a link strength of 21. 24 documents from the Chinese China Medical University Hospital were indexed, with 852 citations and an impressive link strength of 47. Notably, Harvard Medical School in the US produced 21 documents and 202 citations with a link strength of 13, whereas Mayo Clinic and Mayo Foundation, also in the US, produced 21 documents and 866 citations with a link strength of 11, both of which are located in the US. Massachusetts General Hospital, a different American institution, produced 19 documents, racked 1024 citations, and displayed a link strength of 14. These organizations, which represent a variety of nations, highlight their significant presence and collaborative prowess within co-authorship networks, which are distinguished by high document counts, citation impact, and link strength, underscoring their crucial roles in international collaborative research projects (Fig. 3).

### 3.5. Keyword mapping analysis

**Figure 4.**
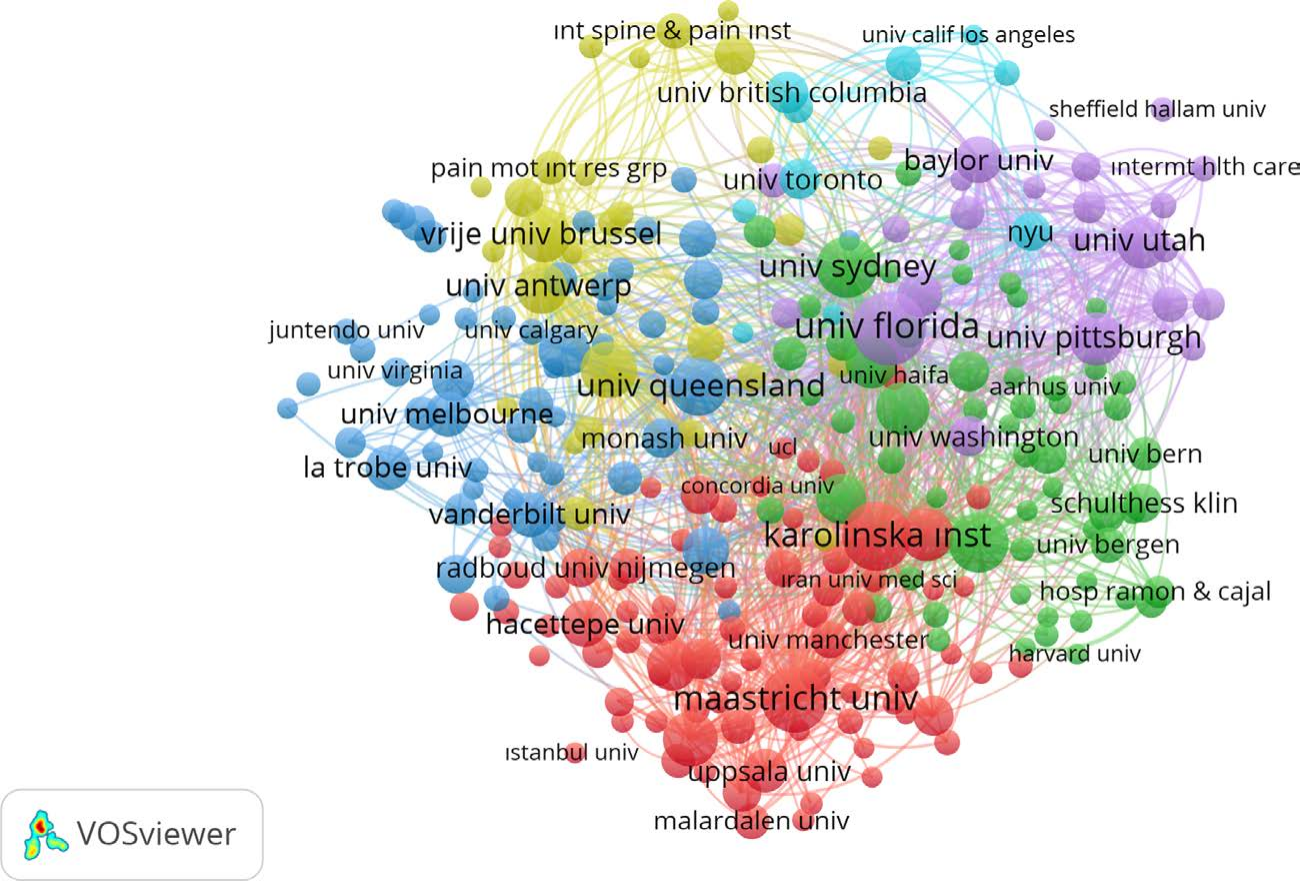
Keyword mapping analysis.

A minimum occurrence threshold of 5 was used in a thorough keyword mapping analysis using Vosviewer, resulting in the discovery of 117 out of 2007 keywords that satisfied the required criteria. Several of these keywords, along with their respective total link strengths, have emerged as well-known and frequently used. The word “chondrosarcoma” was the most common, appearing 618 times, and it had a strong link strength of 902, highlighting its crucial significance in research discussions. The term “Chordoma” came in second with 68 occurrences and a total link strength of 168, while the term “Mesenchymal Chondrosarcoma” was 58 times with a link strength of 55. Additionally, “Extraskeletal Myxoid Chondrosarcoma” was mentioned 50 times, “Skull Base” was mentioned 47 times, “Prognosis” was mentioned 45 times, and “Surgery” was mentioned 44 times. Each of these terms had a different degree of total link strength, but they all collectively reflected important themes and areas of focus for chondrosarcoma research (Fig. 4 and Table 2).

**Table 2:** The top occurred keywords with more than 10 occurrences.

Keyword	Occurrences	Total link strength
Chondrosarcoma	618	902
Chordoma	68	168
Mesenchymal chondrosarcoma	58	55
Extraskeletal myxoid chondrosarcoma	50	49
Skull base	47	124
Prognosis	45	113
Surgery	44	129
Sarcoma	40	81
Radiotherapy	34	96
Metastasis	33	69
Chemotherapy	31	91
Dedifferentiated chondrosarcoma	31	52
Enchondroma	30	62
Apoptosis	29	39
Survival	28	91
MRI	23	43
Immunohistochemistry	22	39
Spine	21	69
Proton therapy	20	41
Clear cell chondrosarcoma	19	28
Case report	17	19
Prognostic factors	17	45
Recurrence	17	38
Treatment	17	39
Pelvis	15	31
Angiogenesis	13	14
Osteochondroma	13	22
Radiation therapy	13	45
Chondrosarcomas	12	13
Hand	12	34
Magnetic resonance imaging	12	25
Outcome	12	40
Stereotactic radiosurgery	12	38
Bone tumor	11	18
Local recurrence	11	27
Nomogram	11	31
Oncology	11	31
Osteosarcoma	11	25
Overall survival	11	41
Tumor	10	24

### 3.6. The analysis of top publishing journals on chondrosarcoma

“Clinical Orthopaedics and Related Research” and “Skeletal Radiology” stand out as significant contributors, each accounting for 3.817% and 3.664% of the total records, respectively, according to the analysis of publishing journals in the context of chondrosarcoma research. Additionally, this group of journals, which includes “Cancer,” “Journal of Orthopaedic Research,” and “Anticancer Research,” have emerged as the most prolific journals. Table 3 summarizes the journals with the highest number of publications on chondrosarcoma and articles.

**Table 3:** Top publishing journals.

Publication titles	Record count	% of 1.310	Category quartile
Clinical Orthopaedics and Related Research	50	3.817	Q1
Skeletal Radiology	48	3.664	Q3
Cancer	37	2.824	Q1
Journal of Orthopaedic Research	27	2.061	Q2
Anticancer Research	26	1.985	Q4
Chordomas and Chondrosarcomas of the Skull Base and Spine, 2nd edition	25	1.908	–**
Oncology Letters	23	1.756	Q3
Journal of Surgical Oncology	22	1.679	Q4
World Neurosurgery	21	1.603	Q4
American Journal of Surgical Pathology	19	1.450	Q1

*Source: Journal Citation Reports 2022.

** E-book, do not contain quartile data.

## 4. Discussion and conclusions

The WOSCC was searched comprehensively for all pertinent articles with a publication date range of January 1, 1993, to September 1, 2023, to gather data for this retrospective bibliometric study. Data analysis produced intriguing patterns. The percentages of 24.504%, 36.489%, and 52.672%, respectively, orthopedics, surgery, and cancer were subjects of a sizable fraction of the publications. A closer look at publications from 3 ten-year periods revealed a considerable increase in research output from 2014 to 2023, accounting for 41.74% of the papers. This increase in research activity and interest in chondrosarcomas points to a thriving and developing research landscape in these specialized fields.

The geographic origins of the research were also examined, with the United States emerging as the top publishing country, closely followed by China and Japan. These findings demonstrate how chondrosarcoma research is a global collaborative effort, with North America, Asia, and Europe emerging as major publishing locations.

The dataset revealed a wide range of notable associations, with the United States leading the pack with organizations such as Harvard University and the Mayo Clinic, displaying their significant contributions to the fields of oncology, surgery, and orthopedics. Associations with institutions such as Leiden University and Leiden University Medical Center serve as evidence of the Netherlands’ strength in research. With associations such as China Medical University Taiwan playing a considerable role, Taiwan has developed into a noteworthy contributor. Affiliations with institutions such as the University of London and University College London serve as examples of the United Kingdom’s presence, while the National Cancer Center Japan serves as a showcase for Japan’s contribution. The broad scope and varied affiliations that influence research in these specialist disciplines are highlighted by international collaboration.

This study provides important new perspectives on chondrosarcoma research and its scholarly significance. First, the high volume of citations (30,076 in total) underscores the significance and applicability of this research field. With an average of 22.96 citations per article and 23,048 total citations (excluding self-citations), chondrosarcoma-related studies have a wide range of applications, highlighting the ongoing importance of this research to the scientific community. Furthermore, the contributions of highly cited articles, such as Amary et al’s work on IDH1 and IDH2 mutations, Gelderblom et al’s clinical approach towards chondrosarcoma, and Damron et al’s exploration of osteosarcoma, chondrosarcoma, and Ewing’s sarcoma, highlight the pivotal role of these studies in shaping the field and providing valuable insights into the understanding, diagnosis, and treatment of chondrosarcoma.

Furthermore, analyses of co-authorship among countries and organizations have demonstrated the collaborative nature of chondrosarcoma research. Leading contributors have emerged from nations, such as the United States, China, Japan, and Italy, demonstrating their significant involvement in cooperative research projects. Likewise, institutions with global influence in the field, such as Leiden University, China Medical University, Mayo Clinic, and Harvard Medical School, have been crucial in fostering collaborative research networks. Overall, this information offers a thorough picture of the global chondrosarcoma research landscape and highlights the cooperative efforts and contributions of scientists, organizations, and nations in furthering our understanding of this complex disease.

In the field of medicine, keyword analysis is crucial because it provides healthcare professionals with access to the most recent studies, clinical recommendations, and treatment modalities, enhancing patient care and diagnosis. Additionally, it supports medical research, assists in the identification of emerging health trends, drug development, and the discovery of novel therapies, furthering our understanding of diseases and enhancing patient outcomes. Keyword analysis is a crucial tool that supports every aspect of modern medicine, ensuring the provision of high-quality healthcare and continuous advancement of medical knowledge.^[11,13,15,17,18]^ The data from the keyword mapping analysis that is being presented has a significant impact on many areas of academic and clinical research. First, it offers insightful information on major themes and areas for future studies in the field of chondrosarcoma and related subjects. The prevalence of terms like “Chondrosarcoma,” “Chordoma,” and “Mesenchymal Chondrosarcoma” highlights the main focus of research initiatives and enables researchers and institutions to focus their resources on topics with high potential for impact. Second, the total link strengths linked to these keywords revealed the degree of interconnectedness of the scholarly landscape, pointing to potential directions for cross-disciplinary collaboration. The data also emphasize the crucial role of terms like “Prognosis” and “Surgery,” emphasizing the significance of clinical outcomes and treatment approaches in addressing chondrosarcoma.

Knowing the target journals with the highest number of publications in a subject may help researchers in that field select a journal to publish their publications.^[19,20]^ In our study, “clinical orthopaedics and related research, a leading peer-reviewed orthopedic journal affiliated with The Association of Bone and Joint Surgeons, played an important role in the publication of chondrosarcoma articles. The journal serves as a critical platform for the dissemination of the latest orthopedic information, including the latest clinical and basic research, and informed opinions that influence contemporary orthopedic practice, thereby promoting evidence-based medicine. With contributions from leading clinicians and researchers worldwide, clinical orthopaedics and related research is dedicated to advancing international understanding and knowledge of musculoskeletal research.”

There are a few limitations to this study that should be considered. First, because it excluded potentially pertinent material from other databases, our search was restricted to entries in the WOSCC database, which may have introduced selection bias. Additionally, our inclusion criteria concentrated on studies in the domains of orthopedics, surgery, and cancer that were written and published in English between 1993 and 2023. This constraint might possibly reduce the breadth of our study by excluding important findings published in other languages or in alternative document formats. Second, it is crucial to acknowledge the changing nature of the scientific processes. New research has been frequently published, and the field of chondrosarcoma is constantly changing. As our study relied on a static dataset from a given time period, there might be discrepancies between the results of our bibliographic analysis and the actual state of research at the time of reading.

Third, there is a possibility that the database’s accuracy and completeness will be questioned. The reliability of our conclusions may be affected by mistakes in document labeling, incorrect document type classification, and possible omissions of pertinent documents. In light of these limitations, researchers should be cautious when interpreting findings and thinking about incorporating data from various sources and languages to obtain a more thorough understanding of the subject.

Furthermore, it is critical to be aware of the possibility of bias resulting from authors with identical names or variations in keyword expressions despite our efforts to standardize the data manually. Consequently, different researchers with similar names might be mistakenly linked to specific research themes or topics, which can introduce variability and inaccuracies in the analysis.

Finally, while useful for bibliometric analysis, using software tools such as VOSviewer has drawbacks. These tools may not capture all pertinent data, and data analysis may contain errors that could lead to discrepancies between the results produced by various software programs. Caution should be exercised when analyzing and relying on the output of such tools.

By providing a thorough assessment of the current state and future directions of the field, this bibliometric analysis closes a significant gap in our knowledge of 30-year chondrosarcoma research trends. Researchers, practitioners, and policymakers can benefit from the data-driven insights discovered in this study to keep up with advancements in the fields of orthopedics, surgery, and cancer related to chondrosarcomas. Overall, this analysis supports the pursuit of advancements in chondrosarcoma research and patient care by directing research, encouraging collaboration, and facilitating evidence-based decision-making.

## Author contributions

**Conceptualization:** Selçuk Yilmaz, Mehmet Kurt.

**Data curation:** Selçuk Yilmaz, Mehmet Kurt.

**Formal analysis:** Selçuk Yilmaz, Mehmet Kurt.

**Funding acquisition:** Selçuk Yilmaz, Mehmet Kurt.

**Investigation:** Selçuk Yilmaz, Mehmet Kurt.

**Methodology:** Selçuk Yilmaz, Mehmet Kurt.

**Project administration:** Selçuk Yilmaz, Mehmet Kurt.

**Resources:** Selçuk Yilmaz, Mehmet Kurt.

**Software:** Selçuk Yilmaz, Mehmet Kurt.

**Supervision:** Selçuk Yilmaz, Mehmet Kurt.

**Validation:** Selçuk Yilmaz, Mehmet Kurt.

**Visualization:** Selçuk Yilmaz, Mehmet Kurt.

**Writing – original draft:** Selçuk Yilmaz, Mehmet Kurt.

**Writing – review & editing:** Selçuk Yilmaz, Mehmet Kurt.
